# Catheter-directed thrombolysis versus percutaneous mechanical thrombectomy in the management of acute limb ischemia: a single center review

**DOI:** 10.1186/s42155-018-0041-1

**Published:** 2018-12-03

**Authors:** Ravi Kumar Muli Jogi, Karthikeyan Damodharan, Hing Lun leong, Allison Chek Swee Tan, Sivanathan Chandramohan, Nanda Kumar Karaddi Venkatanarasimha, Farah Gillan Irani, Ankur Patel, Apoorva Gogna, Kiang Hiong Tay, Thijs August Johan Urlings

**Affiliations:** 1 Singapore General Hospital, Hospital drive, Singapore, 169608 Singapore; 2 Perth, Australia

**Keywords:** Acute limb ischemia, Thrombectomy, Thrombolysis

## Abstract

**Background:**

Acute limb ischemia is associated with significant mortality and amputation rate. Early restoration of flow can be obtained by various treatment methods that include catheter-directed thrombolysis (CDT) and percutaneous mechanical thrombectomy (PMT). These treatments have been shown to be effective but associated with various complications. There is lack of data comparing these two treatments. We aim to review our experience in the treatment of acute limb ischemia (ALI) and compare CDT with PMT.

**Results:**

A total of 94 patients [mean age 65 years, 67% male (*n* = 63)] presented with ALI between 2006 and 2015 and were treated with either CDT or PMT. Outcomes were retrospectively reviewed. Primary outcomes were technical and clinical success; secondary outcomes were amputation rate at 30 days, duration of hospitalization and 30-day mortality. A total of 117 procedures were performed in 94 patients: 27 surgical bypass grafts, 31 previously stented arteries and 59 native vessels. Twenty eight procedures (24%) were performed with PMT, and 89 (76%) procedures were performed with CDT. Higher technical success was achieved in the PMT group (68%, 19/28) compared to the CDT group (47%, 42/89), *p* = 0.056. Clinical success was similar in both groups (75%, 21/28 in the PMT group and 73%, 65/89) in the CDT group (*p* = 0.837). There was no statistically significant difference in 30-day mortality between the PMT vs CDT groups (4% vs 8%, *p* = 0.425). The length of post-procedural hospital stay was shorter in patients with PMT (6.0 vs 12.6 days, *p* = 0.001). The absence of end-stage renal failure appears to be a predictor for clinical succes (HR 3.3, 95% CI 0.809–13.592).

**Conclusion:**

PMT is associated with higher technical success and significantly shorter length of stay compared to CDT; however, clinical success is similar across both treatment entities. The safety profile is comparable.

## Introduction

Acute limb ischemia (ALI) is defined as a sudden significant decrease in limb perfusion that has been present for less than 14 days that is causing a threat to limb viability (Patel et al., 2003). To preserve viability and to reduce mortality and morbidity, early restoration of blood flow is crucial. This can be achieved by various surgical or endovascular techniques depending on the severity (Norgren et al., 2007).

Catheter directed thrombolysis (CDT) has been the first line treatment for acute limb ischaemia since the STILE and TOPAS trials in the 1990s (The STILE investigators, 1994; Ouriel et al., 1996). CDT is currently indicated in patients who do not have contraindications and who fall under I and IIA-IIB Rutherford classification categories (Byrne et al., 2014) for ALI. CDT requires long infusion times and close monitoring in an intensive care unit. Major risks of CDT include hemorrhage, including up to a 0.4% risk of potentially fatal intracranial haemorrhage when treated with Urokinase (Karnabatidis et al., 2011). Despite treatment, ALI is still associated with significant mortality (9–15%) and an amputation rate reaching 30% (Dormandy et al., 1999).

Due to these associated risks, percutaneous mechanical thrombectomy (PMT) devices have gained popularity. Some studies have supported the use of PMT as a first- line treatment option for ALI because this can reduce the procedure time and rapidly improve perfusion (Leung et al., 2015a).

We aim to compare the efficacy and safety profile of CDT and PMT in the management of acute limb ischemia.

## Materials and methods

This is a retrospective study approved by the institutional review board in a tertiary referral hospital. The data from 117 procedures performed in 94 patients between 2006 and 2015 was collected. Treatment choice (CDT or PMT) in every case was based on the clinical scenario and the operator’s expertise in PMT use.

Primary outcomes were technical and clinical success.

Technical success was defined as removal of 99% of clot.

Clinical success was defined as a return to premorbid Rutherford score without major amputation within 30 days.

The use of minor additional techniques to remove clots, such as balloon maceration and aspiration (without dedicated suction catheters), was considered part of the primary procedure in either group. Additional CDT after PMT or vice versa was deemed technical failure. The use of protective filter was not part of the treatment.

Secondary outcomes were amputation rate at 30 days, duration of hospitalization and 30-day mortality. The complications were recorded according to the CIRSE classification of complications (Filippiadis et al., 2017).

### Treatment

CDT was performed according to the department protocol by infusing Urokinase (China chemicals, Hsinchu, Taiwan) via the infusion catheter in the thrombus (Craig McNamara catheter, Medtronic, USA). The typical dose of Urokinase is 2000 units/minute (120,000 units/hour). Simultaneously, heparin was infused intravenously starting at a rate of 800 units/hour. The patient returned to the angiography suite after 6 h for assessment and further intervention if needed.

ROTAREX (Straub Medical, Wangs, Switzerland) and ANGIOJET (Boston Scientific, Massachusetts, USA) were the two mechanical thrombectomy devices used at our institution during the study period. ANGIOJET was first used in power pulse mode using 120,000–240,000 units of Urokinase before switching to thrombectomy mode. Adjunctive procedures such as angioplasty were performed as appropriate.

### Statistical analysis

Pearson’s Chi-square test was used to analyze the categorical data, and a T-test was used to analyze the continuous data (*P* < 0.05 was taken to be significant). Binary logistic regression was performed to identify predictors for technical and clinical success (*p* value < 0.2 was taken to be significant in univariate analysis).

Multivariate logistic regression was performed, done for the significant predictors from the univariate analysis (a *P* value < 0.05 was taken to be significant).

## Results

A total of 117 procedures were performed in 94 patients [mean age 65 years, 67% male (*n* = 63)]. Of the 117, 27 were surgical bypass grafts, 31 were previously stented arteries and 59 were native vessels. 28 procedures (24%) utilized PMT, and 89 (76%) procedures utilized CDT. Of the 28 procedures in the PMT group, 10 were performed with ROTAREX and 18 were performed with ANGIOJET.

Both groups were comparable in terms of age, gender and co-morbidities (see Table 1).

**Table 1:** Population characteristics

	PMT (*n* = 28)	CDT (*n*=89)	p value
Mean age +/− SD	63.3 +/− 10.5	66.0 +/− 12.4	0.310
Male gender	18 (64.3%)	60 (67.4%)	0.759
AGE GROUP			
30–50 years	3 (10.7%)	12 (13.5%)	0.382
51–70 years	18 (64.3%)	44 (49.4%)	
71–90 years	7 (25%)	33 (37.1%)	
CO-MORBIDITIES			
Diabetes mellitus	16 (57.1%)	55 (61.8%)	0.660
Hypertension	21 (75%)	67 (75.3%)	0.976
IHD	11 (39.3%)	51 (57.3%)	0.096
CVA	4 (14.3%)	16 (8%)	0.651
Smoking	9 (32.1%)	25 (28.1%)	0.680
ESRF	3 (10.7%)	6 (6.7%)	0.491

The characteristics of clinical presentation and location of the occlusion are presented in Table 2. The duration of symptoms was comparable between both groups; however, there was a tendency for a lower Rutherford classification for ALI in the PMT group [category I and IIA in 82% (23/28)] than the CDT group [63% (56/89)] with a *p* value of 0.058. More native vessels [59.6% (53/89) vs 29% (8/28)] and bypasses [27% (24/89) vs 14% (4/28)] were treated with CDT. By chance, the majority of stented vessels were in PMT group [57% (16/28) vs 13.4% (12/89)]. In the CDT group, 57% (50/89) of patients were treated for femorocrural occlusions compared to 25% (7/28) in the PMT group. In the PMT group, 71% (20/28) received adjunctive angioplasty compared to 64% (57/89) in the CDT group. The average length of occlusion in PMT group was 25 cm and 34.5 cm in CDT group. The mean duration of CDT was 11.9 h with a standard deviation of 8.48. 36% patients (10/28) in PMT group and 31% (28/89) in CDT group had antegrade puncture.

**Table:** Table 2: Clinical presentation and location of occlusion

	PMT (*n* = 28)	CDT (*n* = 89)	*p* value
DURATION OF SYMPTOMS			
Less than 24 h	5 (17.9%)	13 (14.6%)	0.338
1 to 7 days	12 (42.9%)	52 (58.4%)	
8 to 14 days	11 (39.25)	24 (27%)	
RUTHERFORD CLASSIFICATION			
I and IIA	23 (82.1%)	56 (62.9%)	0.058
IIB	5 (17.9%)	33 (37.1%)	
OCCLUSION			
Thrombosed at stent	16 (57.1%)	12 (13.4%)	
Thrombosed at bypass	4 (14.3%)	24 (27%)	
Thrombosed at native vessel	8 (28.6%)	53 (59.6%)	
OCCLUDED			
Iliac	1 (3.6%)	0 (0%)	
Iliofemoral	1 (3.6%)	5 (5.6%)	
Femoral, popliteal	15 (53.6%)	30 (33.7%)	
Femorocrural	7 (25%)	50 (57.3%)	
Crural & pedal	4 (14.3%)	3 (3.4%)	

### Primary and secondary outcomes

Primary and secondary outcomes are presented in Table 3. Higher technical success was achieved in the PMT group (68%, 19/28) compared to the CDT group (47%, 42/89) (*p* = 0.056). However, clinical success was similar between the groups (75%, 21/28 in the PMT group and 73%, 65/8 in the CDT group (*p* = 0.837). PMT was converted to CDT in 14% (4/28) and CDT was converted to PMT in 6% (5/89), which was considered treatment failure.

**Table 3:** Results summary

	PMT (*n* = 28)	CDT (*n* = 89)	*p* value
PRIMARY OUTCOME			
Clinical success	21 (75%)	65 (73%)	0.837
Technical success	19 (67.9%)	42 (47.2%)	0.056
SECONDARY OUTCOME			
Major amputation* (30 days)	2 (7.1%)	15 (16.9%)	0.323
Minor amputation(30 days)	1 (3.6%)	1 (1.1%)	
Hospitalization (mean days)	6.0	12.6	0.001
COMPLICATIONS (CIRSE) (Filippiadis et al., 2017)			
Grade 2	6 (21.4%)	21 (23.57%)	
Grade 3	1 (3.6%)	4 (4.5%)	
Grade 6	1 (3.6%)	2 (2.3%)	
30 Day mortality	1 (3.6%)	7 (8%)	0.425

The major amputation at 30 days was 7.1% (2/28) in the PMT group vs 16.9% (15/89) in the CDT group (*p* = 0.323). The minor amputation rate at 30 days was 3.6% (1/28) in the PMT group vs 1% (1/89) in CDT group. The length of post procedural hospital stay was shorter in patients undergoing PMT (6.0 vs 12.6 days, *p* = 0.001).

### Regression analysis for primary outcomes

A logistic univariate and multivariate regression analysis was performed to identify significant predictors of technical and clinical success. PMT was a significant predictor for a higher technical success (HR 3.2, 95% CI 1.084–9.326, *p* = 0.035). However, the use of PMT was not found to be a significant predictor for clinical success. The absence of end-stage renal failure appears to be a predictor for clinical succes (HR 3.3, 95% CI 0.809–13.592); however, this did not reach statistical significance (*p* = 0.096). End stage renal disease is defined by Kidney Disease Outcome Quality Initiative, as the patient being dialysis dependent (KDOQI guideline).

### Complications

Complications are presented in Table 3. In the PMT group, closure device was used in 48% (14/29). Groin haematoma was seen in 2 patients treated with closure device. 1 patient without closure device had pseudoaneurysm and 1 patient died secondary to a massive groin haematoma. 1 patient had chest wall hematoma requiring transfusion and 1 patient had fasciotomy for compartment syndrome. Distal embolization was seen in 1 patient.

In the CDT group, closure device was used in 45% (40/89). Groin haematoma was seen in 7 patients  with closure device and 7 patients without closure device. Gastro-intestinal bleed requiring transfusion were seen in a total of 7 patients. Compartment syndrome requiring fasciotomy was recorded in 4 patients. There were no cases of intracranial haemorrhage in the CDT group. Two deaths were recorded in this group; one secondary to acute renal failure and adult respiratory syndrome, the other as a complication of compartment syndrome. Distal embolization was seen in 2 patients.

## Discussion

Our study shows higher technical success in the PMT group compared to the CDT group (68% vs 47%). However, CDT in this cohort was used in more complex distal occlusive disease. This is likely because the use of PMT below the knee vessels is thought to be risky due to small calibre vessels in our population. Conversion of one treatment to another was only seen in 14% of patients in PMT group and 6% of patients in CDT group, hence, did not contribute much towards drop in technical success. The choice of treatment is also dependent on the operator’s experience and knowledge of thrombectomy devices. This selection bias is inherent to the retrospective nature of this study.

Few studies have demonstrated high technical success with PMT ranging between 56 and 95% (Karnabatidis et al., 2011). Kasirajan et al. investigated PMT using ANGIOJET in acute and subacute limb ischemia and divided technical success into failure (< 50% luminal restoration), partial success (50–95% luminal restoration) and complete success (> 95% luminal restoration). The complete success rate was 61.4% (Kasirajan et al., 2001).

Similarly, Byrne et al. demonstrated 90% technical success rate but they defined technical success as the restoration of inline flow to the foot without requiring immediate surgical intervention (Byrne et al., 2014), which is similar to our definition. However, we also attempted to quantify the clot burden and defined technical success as the removal of 99% of the clot.

Despite PMT being technically superior, the clinical success remains similar in the PMT and CDT groups (75% vs 73%) and PMT was also not a predictor for clinical success in the regression analysis.

The regression analysis showed that patients without end-stage renal disease had a tendency for higher clinical success. Patients with end-stage renal disease tend to have extensive vascular disease and revascularization can be challenging in such cases. Similar findings were reported by Byrne et al.; they reported a 6 to 26-fold increased risk of amputation and the loss of primary and secondary patency in patients with end-stage renal disease (Byrne et al., 2014).

As previously noted, the major limiting factor for CDT is hemorrhagic complications. Seven percent (*n* = 6) of patients receiving CDT in this study had major bleeding. Of these, 3 patients had groin access hematomas and 2 patients had gastro-intestinal bleeding. Seven percent (*n* = 2) of patients receiving PMT had major groin access hematomas; this difference was not statistically significant. None of the patients receiving CDT had a hemorrhagic stroke in our cohort. Karnabatidis et al. reported a 0.4% risk of potentially fatal intracranial hemorrhage when treated with Urokinase (Karnabatidis et al., 2011).

In this study, there was no significant difference in 30-day mortality between CDT and PMT (8% vs 4%, *p* = 0.425); this is comparable to the published data. Byrne et al. reported 30-day mortality in 4.8% of patients with CDT and 5.6% with PMT (*p* = 0.82) (Byrne et al., 2014).

The duration of hospitalization was significantly shorter in patients who underwent PMT; the mean stay was 6 days in PMT group vs 12.6 days in the CDT group, *p* = 0.0001. PMT was used more in category I and IIa patients with femoral and popliteal disease. The reduced technical success in the CDT group may have played a role in prolonging the hospital stay in this group. The mean duration of CDT was 12 h in this study; hence, this likely did not play a major role in the duration of hospitalization.

The thirty-day amputation rates were not signficantly different between the PMT vs CDT groups (7% vs 17%, *p* = 0.323). Limb salvage rate for PMT in this study was 93%, which was comparable to literature. Ansel et al. reported limb salvage rate of 89% at 1 month following PMT (Ansel et al., 2008).

This study is limited by its retrospective nature and unequal patient numbers between the PMT group and CDT group. However, there is a lack of randomized trials comparing these two treatments and the indications for these treatments in acute limb ischemia are not clear. The difference in costs between CDT and PMT were not evaluated, which may play a role in decision making because the clinical success rates are similar.

## Conclusion

PMT is safe and effective as a primary treatment for ALI with a similar clinical outcome compared to CDT. Several treatment options are available for this patient group and the choice of treatment should still be on a case-by-case basis. Patients requiring quick revascularization (for example Rutherford IIB) and with contraindications for thrombolysis may benefit from PMT. Similarly, CDT can be the treatment of choice in patients with complex crural disease. A combination of these techniques should be considered when appropriate.

## Abbreviations

ALI: Acute limb ischemia
CDT: Catheter directed thrombolysis
CIRSE: Cardiovascular and Interventional Radiological Society of Europe
PMT: Percutaneous mechanical thrombectomy
